# Anti-HIV activity in traditional Chinese medicine: clinical implications of monomeric herbal remedies and compound decoctions

**DOI:** 10.3389/fmed.2024.1322870

**Published:** 2024-08-08

**Authors:** Nannan Zhang, Mengyuan Wang, Ling Gao, Congying Zhang, Xiaoguang Tang, Xianjun Liu, Chunying Bai

**Affiliations:** ^1^Key Laboratory of Research on Human Genetic Diseases at Universities of Inner Mongolia Autonomous Region, School of Basic Medicine, Chifeng University, Chifeng, China; ^2^College of Biology and Food Engineering, Jilin Engineering Normal University, Changchun, China; ^3^School of Chemistry and Life Science, Changchun University of Technology, Changchun, China

**Keywords:** HIV, traditional Chinese medicine, herbs, composite soup, antiviral activity, clinical significance

## Abstract

With the global spread of human immunodeficiency virus (HIV) infection and acquired immune deficiency syndrome (AIDS), the pursuit of potent treatments has ascended as a paramount concern in global healthcare. Traditional Chinese medicine (TCM) has been used for thousands of years in China and other East Asian countries and it offers remedies for an extensive array of ailments, including HIV and AIDS. This review focuses on the clinical significance of single herbs and composite tonics in TCM with antiviral activity against HIV. Initially, the anti-HIV activity of single herbs was analyzed in detail. Many herbs have been shown to have significant anti-HIV activity. The active ingredients of these herbs exhibit their anti-HIV effects through various mechanisms, such as inhibiting viral replication, preventing viral binding to host cells, and interfering with the viral lifecycle. Furthermore, we delved into the clinical significance of HIV-associated formulations provided as a result of Chinese compound prescription. These combinations of herbal ingredients are designed to amplify therapeutic efficacy and minimize adverse effects. Clinical trials have demonstrated the therapeutic benefits of these prescriptions for individuals infected with HIV. The intricate composition of these prescriptions potentially augments their anti-HIV activity through synergistic effects. Additionally, this review underscores the clinical importance of TCM in the context of HIV treatment. While numerous herbs and prescriptions exhibit anti-HIV activity, their safety and efficacy in clinical applications warrant further investigation. When combined with contemporary antiretroviral drugs, TCM may serve as an adjunctive therapy, assisting in reducing side effects, and enhancing patients' quality of life. To optimally harness these natural resources, further exploration is imperative to ascertain their efficacy, safety, and optimal utilization, thereby offering a broader spectrum of therapeutic options for HIV-afflicted individuals.

## 1 Introduction

Acquired immunodeficiency syndrome (AIDS), a potentially fatal infectious ailment, is caused by the human immunodeficiency virus (HIV), which results in a debilitating compromise of the host's immune system. Consequently, a cascade of opportunistic infections or malignancies ensues, ultimately resulting in mortality for those afflicted (1, 2). The Joint United Nations Programme on HIV/AIDS predicted that, by the end of 2022, 39 million patients would be affected with HIV/AIDS globally, with 1.3 million novel HIV infections reported during that year (3).

The primary targets of HIV are CD4+ T lymphocytes, which play a pivotal role in the immune response against microbial antigens (4). Following viral invasion, the envelope protein Gag, specific to the first domain of CD4 surface marker molecules, orchestrates an onslaught against CD4+ T cells, precipitating a precipitous decline in their numbers (5). Consequently, HIV embarks on a brief but potent replication period, initiating early damage to the host immune system. Owing to its swift progression, formidable challenges, grave repercussions, and wide-ranging effects, HIV/AIDS has emerged as a pivotal public health challenge.

Acquired immunodeficiency syndrome is an immunological disease that poses an immense global threat to human wellbeing. Although conventional highly active antiretroviral therapy (HAART) has achieved remarkable progress in viral suppression and immune augmentation, its side effects and long-term therapeutic impact remain non-negligible. Against this backdrop, traditional Chinese medicine (TCM), which has a venerable history in healthcare systems, is gaining increasing recognition. In the treatment of AIDS, TCM therapies have demonstrated the potential to alleviate clinical symptoms, enhance immune function, and prolong the onset of increased severity of the disease. As a result, research institutions and experts have begun to delve deeper into TCM therapies for HIV, adopting rigorous scientific methodologies to probe its pharmacological mechanisms, efficacy, and possible side effects. Several herbal remedies have yielded positive results in clinical practice, providing useful case studies on the application of herbal medicines in HIV therapy.

Recent advances into herbal treatments for HIV have included a series of breakthroughs, encompassing the screening of diverse herbs and the identification of antiviral active constituents. Herbal screening has increasingly focused on discovering plants that exhibit pronounced anti-HIV activity. Through systematic pharmacological evaluations and laboratory research, several herbal extracts of plants have been identified as potent HIV inhibitors. These herbal extracts likely suppress viral replication and invasion mechanisms. Furthermore, some studies have unveiled the potential of herbs to inhibit key enzyme activities, such as HIV reverse transcriptase. Identification of active constituents is a pivotal aspect of herbal medicine research as it provides a more precise understanding of anti-HIV mechanisms.

Moreover, herbal medicines have the potential for immune modulation; HIV infection severely undermines the immune system of the body, rendering it ineffective against viruses. Hence, studies have explored the potential of herbs to enhance immune function. Some herbs augment immune cell activity, stimulate cytokine secretion, facilitate the restoration of immune homeostasis, and bolster resistance. In addition to laboratory investigations, the number of clinical trials investigating the efficacy of herbal medicines in HIV treatment has increased. Herbal formulations have been integrated into clinical practice, resulting in positive outcomes. Researchers have evaluated the effects of herbal medicines in randomized controlled trials to assess the improvements in clinical symptoms, CD4+ T cell counts, and viral load control. These endeavors have engendered novel perspectives and possibilities for the application of herbal medicines in HIV therapy.

## 2 Monomeric herbal remedies and extracts for HIV treatment

**Figure 1:**
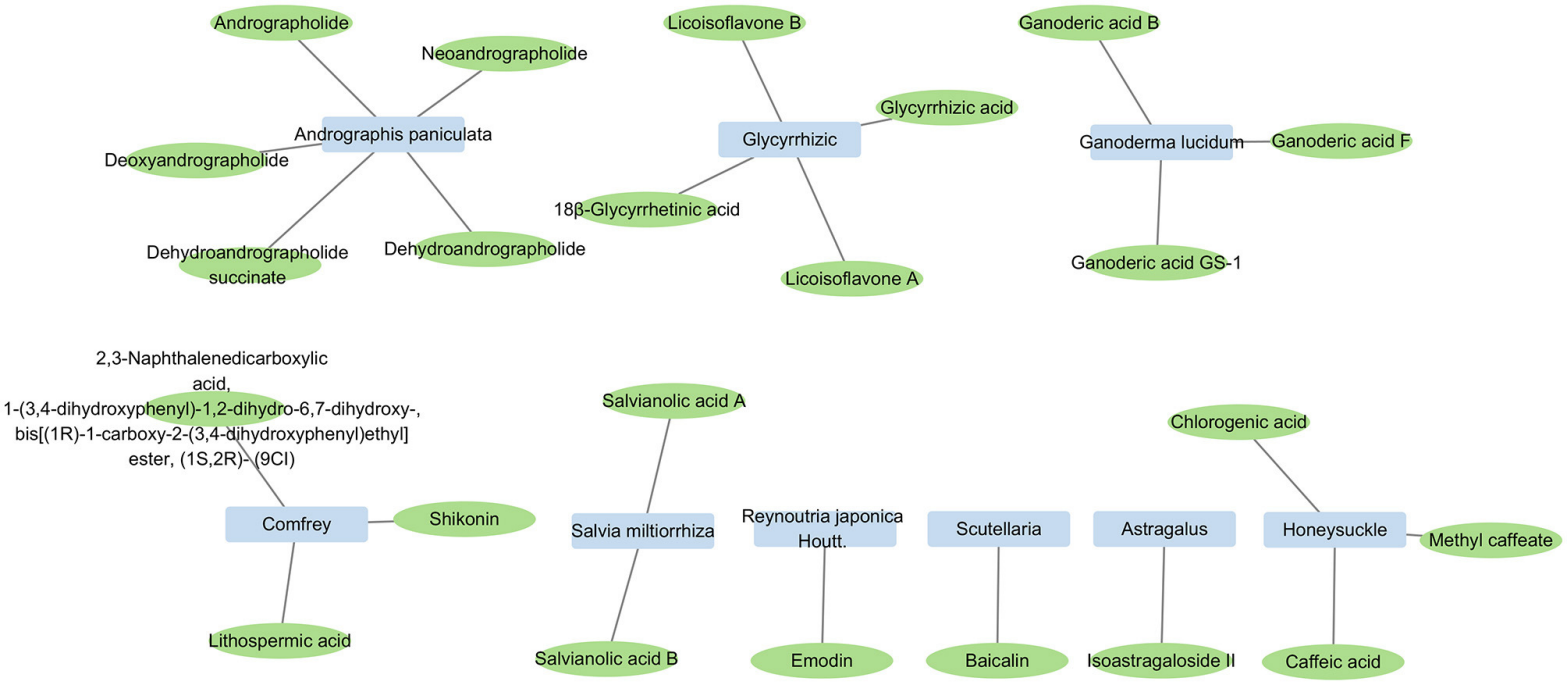
Constituents with anti-HIV activity that are present in single herbs.

Traditional Chinese medicine derives its ingredients from various sources, including plants, animals, and minerals; of these, plants are the most commonly used. Plant-derived medicines are the most extensively researched category in anti-HIV therapies within TCM. The active chemical components of notable anti-HIV herbs in TCM are listed in Figure 1.

### 2.1 Licorice

Licorice, a solitary herbal remedy within TCM, is commonly used as an HIV treatment in clinical settings, featuring prominently as an additive in herbal formulations. The effective chemical constituents of licorice, such as glycyrrhizic acid, glycyrrhizin, glycyrrhetinic acid, and their derivatives exhibit a pronounced ability to inhibit viral replication and effectively eliminate dormant viruses, thus demonstrating a broad-spectrum antiviral effect (6). Glycyrrhizin is primarily found in the roots of licorice and has immune-activating properties (7). Glycyrrhizin potentially lowers membrane fluidity and inhibits intercellular viral infections. Harada (8) demonstrated that cells treated with glycyrrhizin exhibit time-dependent increases in membrane fluidity and sensitivity to infection and fusion when placed in a glycyrrhizin-free replacement culture medium, providing evidence for a close association between membrane fluidity and viral infection. Sasaki et al. (9) found that glycyrrhizin induced β-chemokine production in peripheral blood mononuclear cell (PBMC) cultures from patients infected with HIV, thus inhibiting the entry of NSI-HIV. Ninety percent of HIV replication was inhibited in 31% of the samples. Researchers from China (10) discovered that glycyrrhizin supplementation (100 mg/kg/day) protected rats against R5-type HIV-1 gp120 damage by safeguarding rat myocardial function while maintaining normal rat body weight, lipid metabolism, and antioxidant metabolism. Japanese researchers (11) have extracted phenolic components from glycyrrhizin, which enhance its antagonistic effect against HIV in ATL-IK cells. Two novel glycyrrhizin derivatives inhibited HIV proliferation at low concentrations. In addition, a neutral water-soluble polysaccharide, glycyrrhiza polysaccharide (GPS), was isolated from the glycyrrhiza residue and exhibited a 36.2% inhibition rate against HIV (12).

### 2.2 *Arnebia euchroma*

Studies since the 1990s revealed that *Arnebia euchroma*, an important medicinal plant in Xinjiang, exhibited robust anti-HIV activity. Kashwada et al. (13) established the potent anti-HIV-1 activity of hot-water extracts from dried *A. euchroma* roots, especially a 70% aqueous ketone extract, which displayed effectiveness against HIV replication in H9 cells during acute infection. Sodium and potassium salts of caffeic acid tetramers from *A. euchroma* were identified as active components. Chen et al. (14) discovered that Shikonin inhibits chemokine-induced leukocyte migration and monocyte chemotaxis while reducing leukocyte calcium influx. Shikonin significantly downregulated the mRNA expression of the HIV-1 co-receptor CCR5, inhibiting HIV-1 replication, suggesting that the anti-HIV-1 action of Shikonin is related to interference with chemokine receptor expression and function. Maierdan et al. (15, 16), extracted a water-soluble component from *A. euchroma* that protected MT-2 cells against HIV-1 infection and inhibited HIV-1 integrase (HIV-1 IN) activity. *A. euchroma* polysaccharide, another active component, possesses immune-enhancing properties and enhances the diminished proliferative response of splenic lymphocytes in immunosuppressed mice, having immunomodulatory effects (17).

### 2.3 *Isatis tinctoria*

*Isatis tinctoria*, known for its pronounced immune-regulating and broad-spectrum antiviral effects, began to garner research attention in the late 20^th^ century. Chang and Yeung (18) demonstrated that extracts from *I. tinctoria* completely inhibited HIV growth in H9 cells at sub-toxic concentrations, establishing that they can effectively block HIV replication in a dose-dependent manner. Ngan et al. (19) found that dimethyl sulfoxide (DMSO) extracts of *I. tinctoria* effectively inhibited HIV-1 *in vitro*. *I. tinctoria* extracts did not induce interferons or inactivate extracellular HIV or herpes simplex virus; they specifically inhibited HIV but not herpes simplex virus. These studies reported the anti-HIV activity of *I. tinctoria*; however, failed to identify the active compounds. Wang et al. (20) characterized cyclic peptides in *I. tinctoria* and identified five novel and three known cyclic peptides. Some of these peptides displayed anti-HIV activity, with cyclolinopeptide Y5 being the most effective according to testing with an XTT-based *in vitro* anti-HIV assay.

### 2.4 Honeysuckle

The antiviral efficacy of honeysuckle has been validated in numerous experimental and clinical studies. Compounds isolated from honeysuckle, such as 3,5-O-dicaffeoylquinic acid and 3,5-O-dicaffeoylquinic acid methyl ester, exhibit strong inhibitory effects on HIV-1 reverse transcriptase and HDNAP-γ. At concentrations 1–6 μM, all dicaffeoylquinic acids inhibited HIV-1 replication in T-cell lines (21). Other studies have identified 13 additional caffeoylquinic acids, caffeic acids, and caffeic acid methyl esters in honeysuckle, all of which have demonstrated antiviral activity against respiratory viruses (22).

### 2.5 *Astragalus*

*Astragalus*, renowned for its wide-ranging antiviral and immunomodulatory properties (23, 24), contains compounds such as astragaloside, astragaloside IV, and astragalus polysaccharide, all of which have antiviral effects. Studies on the inhibitory effects of astragaloside on HIV-1 demonstrated that both astragaloside and isoastragaloside I have an inhibitory effect on HIV-1 infection. At a concentration of 50 g/mL, isoastragaloside I exhibited noticeable inhibition of virus infection that was lower than the effective concentration of astragaloside I at 100 g/mL. Furthermore, astragaloside and isoastragaloside I inhibit the integration of viral DNA into the host cell genome (25).

### 2.6 Huangcen

Huangcen is a popular TCM material that is used to treat HIV. Studies on the antiviral effects of Huangcen have focused on its molecular mechanism (26). Huangcen extract contains two flavonoid compounds, baicalein and baicalin, which inhibit HIV infection and replication (27). They suppress various stages of HIV-1 replication, including viral entry into cells, viral protease activity, and reverse transcriptase activity (28). With more than 90% inhibition when present at 2 μg/mL, baicalein was the first compound to be reported to effectively inhibit reverse transcriptase activity of murine leukemia virus and HIV. Baicalein exhibited high specificity for reverse transcriptase (29). According to Chen (30), baicalein inhibited HIV-1 in H9 cell cultures with chronic HIV-1 infection, suppressing the activity of HIV-1 reverse transcriptase and cytopathic effects as well as inhibiting viral immunofluorescent and P24 antigens. Li et al. (31) discovered that the anti-HIV-1 activity of baicalin was also observed in primary human PBMC cultures infected with HIV-1 *in vitro* and that baicalin significantly inhibited the replication of HIV-1 in a dose-dependent manner (32). Furthermore, Li et al. (33) found that baicalein at non-cytotoxic concentrations inhibited the fusion of cells expressing CD4+/CXCR4 or CD4+/CCR5 with X4 and R5 HIV-1 Env proteins, respectively. Additionally, baicalein blocked the initial stage of HIV-1 adsorption, preventing the early termination of DNA replication within cells. In a study by Wang et al. (34), the *in vitro* anti-HIV-1 activity of the baicalein-zinc (BA-Zn) complex was compared with that of baicalein and BA-Zn. The results showed that, compared to those of baicalein, BA-Zn had lower cytotoxicity and higher anti-HIV-1 activity. Using electrochemical methods, Sun et al. (35) studied the interaction between baicalein and DNA and speculated that baicalein binds to DNA through intercalation.

### 2.7 *Salvia miltiorrhiza*

*Salvia miltiorrhiza* is a widely used TCM material. In 1996, Chen et al. (36) discovered that *S. miltiorrhiza* extracts had anti-HIV and anti-HBV activities. The water-soluble components of *S. miltiorrhiza* are its major active ingredients, which contain more than 20 distinct monomeric compounds. All compounds possess strong antioxidant properties, with the highest content and activity found in salvianolic acid A and salvianolic acid B (37). Abd-Elazem et al. (38) isolated and purified two water-soluble compounds, M522 and M532, from *S. miltiorrhiza*. High concentrations of these compounds exhibited no cytotoxicity in H9 cells and they were not able to prevent HIV from entering H9 cells or inhibit reverse transcriptase activity in infected cells. However, they strongly inhibited acute HIV-1 infection in H9 cells. Qin et al. (39) isolated four compounds from *S. miltiorrhiza*, including a novel compound and demonstrated anti-HIV activity of the compounds against HIV-1 integrase and HIV-1 reverse transcriptase. Zhang et al. (40) discovered three novel polyphenolic derivatives in *S. miltiorrhiza* water extracts, tested their anti-HIV-1 activity by inhibiting the P24 antigen in MT-4 cells that were infected with HIV-1, and tested their HIV-1 reverse transcriptase activity. Mori et al. (41) found that lithospermic acid, derived from *S. miltiorrhiza*, exhibited strong inhibitory effects on nucleocapsid protein (NC) without spontaneous oxidation. Given that HIV-1 NC is an ideal target for antiretroviral therapy, these results position it as a promising starting point for developing highly effective NC inhibitors.

### 2.8 Herba Andrographis

Herba Andrographis is a TCM material with notable anti-inflammatory and antiviral properties. Current research on Herba Andrographis primarily focuses on its components, including lactones, neochuanxinlian lactones, deoxychuanxinlian lactones, and dehydrochuanxinlian lactones (42). Dehydrochuanxinlian lactone succinate (DASM), isolated from Herba Andrographis lactones, inhibited HIV *in vitro*. In a previous study, DASM prevented HIV-induced cell fusion and HIV binding to H9 cells (43). Ma et al. (44) discovered that rat serum containing Herba Andrographis reduced the activity of CXCR4 and CCR5 promoters in H9 cultured cells *in vitro*. In human studies, Herba Andrographis reduced the surface expression of CXCR4 and CCR5 in peripheral blood CD4+ T lymphocytes, indicating its anti-HIV-1 activity. Mayur et al. synthesized a series of derivatives of Herba Andrographis lactones. One compound (compound 9) displayed a high therapeutic index and inhibited HIV-1 infection by blocking gp120-CD4+/CXCR4/CCR5 interactions. These compounds have been suggested to bind to the V3 loop of the HIV-1 envelope protein gp120. All these studies demonstrated the potential of Herba Andrographis and its derivatives as anti-HIV drugs (45).

### 2.9 *Ganoderma*

*Ganoderma* has been used for medicinal purposes for more than 2,000 years. *Ganoderma* is assumed to possess immunomodulatory, antiviral, anti-tumor, anti-aging, and hypoxia tolerance-enhancing activities (46, 47). In addition to *Ganoderma* extracts, polysaccharides and triterpenoids have been the subject of studies on *Ganoderma*'s antiviral properties. Kim et al. (48) extracted two components of different molecular weights from *Ganoderma* fruiting bodies and observed that the low-molecular-weight component strongly inhibited the cytopathic effects of HIV-1 on CEM cells. El-Mekkawy et al. (49) isolated multiple triterpenoid compounds from methanol extracts of *Ganoderma* fruiting bodies. Among these compounds, ganoderiol F and ganodermanontriol exhibited anti-HIV-1 activity. Both compounds had inhibition concentrations of 7.8 μg/mL, while ganoderic acid B, ganoderiol B, ganoderic acid C1, 3β-5α-dihydroxy-6β-methoxyergosta-7,22-diene, ganoderic acid α, ganoderic acid H, and ganoderiol A showed moderate inhibitory activity against HIV-1 protease, with an IC_50_ of 0.17 ± 0.23 mM. Min et al. (50) isolated triterpenoid compounds, including Ganodermaol A and ganoderic acid β, from *Ganoderma* fruiting bodies and noticed that ganoderic acid β, lucidumol B, ganoderic acid C1, 3β-5α-dihydroxy-6β-methoxyergosta-7,22-diene, ganoderic acid α, ganoderic acid H, and ganoderiol A exhibited significant anti-HIV-1 protease activity with IC_50_ values ranging from 20–90 μM. Devinna et al. (51) investigated the reaction of *Ganoderma* triterpenoid compounds with HIV-1 aspartic proteinase and fibrinolysin I and observed that ganoderic acid B showed the highest affinity for HIV-1 proteinase, further confirming the anti-HIV activity of Ganoderma. Cheng et al. (52) found that *Ganoderma* antler-shaped fruiting bodies had a higher inhibition percentage (54.3% ± 6.2%) and higher cytotoxicity (CC_50_ <300 ppm) compared to *Ganoderma* crude extract at 150 ppm.

### 2.10 *Polygonum cuspidatum*

*Polygonum cuspidatum* is used in a wide range of clinical applications in TCM, with its rhizomes as the key medicinal component. Recently, notable progress has been made in understanding its pharmacological activities, including its lipid-lowering, anti-inflammatory, anti-shock, antioxidant, anti-HIV-1, antimicrobial, and hepatoprotective effects. Schinazi et al. (53) demonstrated that anthraquinone compounds found in *P. cuspidatum* possess anti-HIV activity, with emodin exhibiting an IC_50_ of 36.3 μmol/L against HIV-1. Jiang (54) infected MT-4 cells with HIV and then treated them with a *P. cuspidatum* water extract to observe HIV-1 proliferation. Continuous mixing of infected cells with uninfected cells, along with the addition of the *P. cuspidatum* water extract, completely inhibited giant cell formation. These results suggest that the extract acts on the surface of the virus to prevent its attachment to the cells. The water extract of *P. cuspidatum* had an inhibitory effect on the binding of recombinant gp120 to CD4, suggesting that one of the mechanisms of its anti-HIV-1 activity is the prevention of virus attachment to cells. Additionally, Jiang et al. (55) found that a 10% *P. cuspidatum* water extract effectively inhibited the binding of HIV-1 surface gp120 to CD4 on cell surfaces *in vitro*, partially inhibiting splenomegaly, immune suppression, and viremia in AIDS mice. Furthermore, *P. cuspidatum* extract was demonstrated to significantly inactivate HIV-1 reverse transcriptase *in vitro*, suggesting that its antiviral effect may be related to enzyme inactivation. According to Lin et al. (56), a 70% ethanol extract of *P. cuspidatum* had an inhibitory effect on HIV-1-induced syncytium formation *in vitro* at non-cytotoxic concentrations, with an EC_50_ of 13.94 ± 3.41 μg/mL.

### 2.11 *Prunella vulgaris*

*Prunella vulgaris* is a commonly used TCM in clinical practice that exhibits antiviral and immunosuppressive activities. It contains various chemical components beneficial for human health and its active ingredients have been the subject of increasing research. Yao et al. (57) found that a purified *P. vulgaris* extract effectively inhibited HIV-1 replication in the lymphocyte cell line MT-4, monocyte cell line U937 and peripheral blood monocytes at concentrations of 6, 30, and 12.5 μg/mL, respectively. The extract also blocked cell-to-cell transmission of HIV-1 and interfered with the binding of HIV-1 and purified gp120 to CD4. Overall, the purified *P. vulgaris* extract antagonizes HIV-1 infection in susceptible cells by preventing the virus's attachment to the CD4 receptor. According to Kageyama et al. (58), *P. vulgaris* extract inhibits HIV reverse transcription replication *in vitro* and the active components can be detected in the plasma after oral administration, demonstrating its feasibility for oral administration. Liu et al. (59) screened *P. vulgaris* extracts from various traditional Chinese herbs and found that they exhibit strong antiviral activity and effectively inhibit HIV-1 gp41 six-helix bundle activity. Zhang (60) studied HIV-1-infected PBMCs induced with *P. vulgaris* polysaccharides and investigated their levels of apoptosis and the cytokines IL-2 and IL-4. The IL-2 levels in HIV-infected PBMC were significantly lower than those in the healthy control group, indicating that *P. vulgaris* polysaccharides could increase IL-2 levels in HIV-infected individuals and partially correct abnormal immune function. Oh et al. (61) found that, compared to the ethanol extract, the water extract of *P. vulgaris* exhibited stronger antiviral activity, and the inhibition of the HIV-1 virus mainly occurred through interference with early and post-virus binding events, suggesting that these extracts have potential as antiviral agents against HIV-1.

Since the extensive research of developing anti-HIV drugs has been conducted, more and more TCM have been found to have significant or potential treatment efficacy. Among them, the extracts or active monomer components of TCM have been reported to have various effects; the mechanisms of these effects have mainly been attributed to inhibiting HIV transcription and replication, blocking HIV binding to host cells, or reducing the expression of CD4+ T cell surface receptors. Although there is a lot of research in this field, few reports have compared the effects of different TCM extracts or active monomer components, which will become the focus of future research. Researchers are more concerned about the effects of mixture which consist of different TCM extracts or active components. Perhaps they believe that using them alone may not be fully functional, and the mixing effects are better, which is also in line with the concept of TCM compatibility. Below, we will discuss the research on mixed medication.

## 3 Therapeutic efficacy of compound decoctions on HIV

**Figure 2:**
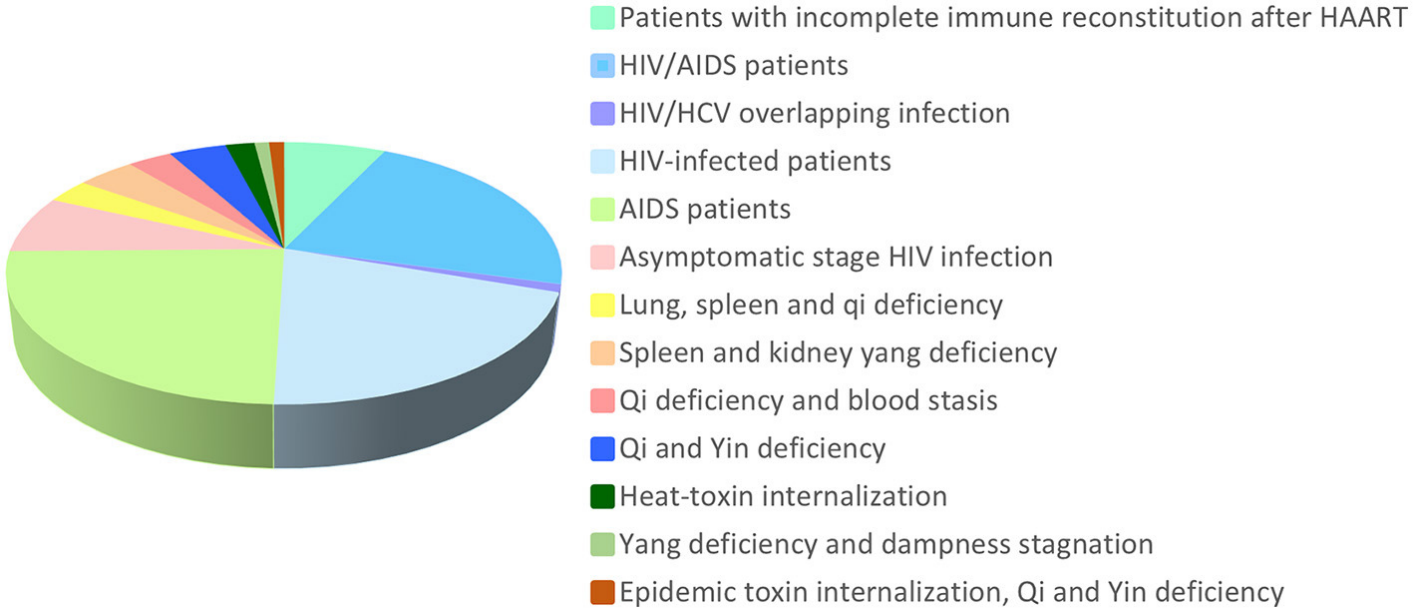
Distribution of the duration of clinical diagnosis for the study population.

Compound decoctions, a form of TCM herbal preparations, have garnered attention recently for the treatment of patients with HIV/AIDS. Several clinical trials have assessed the efficacy of compound decoctions in controlling HIV infection, improving immune function, and enhancing the quality of life of patients. These clinical trials typically employ a double-blind, randomized, placebo-controlled approach in which patients are divided into groups receiving compound decoctions and placebo. The observations included changes in clinical symptoms, viral load, CD4+ T lymphocyte counts, and other relevant indicators. Preliminary findings suggest that some compounded tonics may be more effective in suppressing HIV, improving immune function of patients and alleviating symptoms. Clinical data on compounded tonics that have been shown to be effective in suppressing HIV are listed in Table 1. The distribution of the duration of the clinical diagnosis in the study population was also determined (Figure 2).

**Table 1:** Clinical data on compounded tonics with a more significant effect on HIV viral suppression.

**Drug name**	**Combined Use?**	**Year**	**Population size**	**Treatment group size**	**Dosage**	**Lymphocyte detection**	**HIV viral load detection**	**Anti-HIV mechanism**	**Drug ingredients**	**References**
Yi Ai Kang Capsules	-	2010	885	885	5 pills each time, 3 times daily	CD4+	Viral load decreased and stabilized in 74.25% of cases	Nourishes Qi, strengthens spleen, nourishes blood, dispels wind and clears heat, dries dampness and detoxifies	Ginseng, Astragalus, Fried Atractylodes, Poria, Angelica, Ligusticum, Peony, Scutellaria	(62)
Yi Ai Kang Capsules	-	2008	379	379	5 pills each time, 3 times daily	CD4+	Viral load decreased and stabilized in 84.9% of cases	Significantly stabilizes or slows the decline of CD4+ T lymphocytes and reduces or stabilizes viral load through other mechanisms	Ginseng, Astragalus, Fried Atractylodes, Poria, Angelica, Ligusticum, Peony, Scutellaria	(63)
Yi Ai Kang Capsules	Yes	2022	238	119	5 pills each time, 3 times daily	CD4+ and CD8+	-	Enhances the patient's CD4T lymphocyte count and strengthens immune function	Ginseng, Astragalus, Fried Atractylodes, Poria, Angelica, Ligusticum, Peony, Scutellaria	(64)
Yi Ai Kang Capsules	-	2022	60	20	5 pills each time, 3 times daily	CD3+, CD4+, CD8+, CD4+/CD8+ ratio	-	-	Ginseng, Astragalus, Fried Atractylodes, Poria, Angelica, Ligusticum, Peony, Scutellaria	(65)
Yi Ai Kang Capsules	Yes	2022	51	34	5 pills each time, 3 times daily	CD4+, CD8+, CD4/CD8, CD45RO+, CD45RA+	-	Nourishes spleen and Qi, nourishes blood, clears heat, and dispels wind	Ginseng, Astragalus, Fried Atractylodes, Poria, Angelica, Ligusticum, Peony, Scutellaria	(66)
Yi Ai Kang Capsules	-	2022	18	18	5 pills each time, 3 times daily	-	-	-	Ginseng, Astragalus, Fried Atractylodes, Poria, Angelica, Ligusticum, Peony, Scutellaria	(67)
Yi Ai Kang Capsules	-	2018	75	65	5 pills each time, 3 times daily	-	-	-	Ginseng, Astragalus, Fried Atractylodes, Poria, Angelica, Ligusticum, Peony, Scutellaria	(68)
Yi Ai Kang Capsules	-	2018	20	20	5 pills each time, 3 times daily	-	-	-	Ginseng, Astragalus, Fried Atractylodes, Poria, Angelica, Ligusticum, Peony, Scutellaria	(69)
Yi Ai Kang Capsules	-	2017	48	-	5 pills each time, 3 times daily	-	-	-	Ginseng, Astragalus, Fried Atractylodes, Poria, Angelica, Ligusticum, Peony, Scutellaria	(70)
Aikeqing Capsules	-	2010	44	44	3 pills each time, 3 times daily	-	-	-	Epimedium, Ligustrum, Baical skullcap, Salvia, Polygonum, Licorice, Astragalus, Processed Aconite, Dried Ginger	(71)
Aikeqing Capsules	Yes	2017	69	47	3 pills each time, 3 times daily	CD4+ and CD8+	-	Beneficial for spleen and kidney, clears heat and detoxifies, especially beneficial for HIV/AIDS patients with spleen and kidney damage, lingering pathogenic factors. Epimedium can activate macrophages and elevate white blood cells. Huangqin, Polygonum, and Salvia can clear heat, detoxify, promote blood circulation, and relieve pain. Licorice enhances cell-mediated immunity	Epimedium, Ligustrum, Baical skullcap, Salvia, Polygonum, Licorice, Astragalus, Processed Aconite, Dried Ginger	(72)
Aikeqing Capsules	Yes	2009	60	30	3 pills each time, 3 times daily	CD4+	Viral load reduction not significant	-	Epimedium, Ligustrum, Baical skullcap, Salvia, Polygonum, Licorice, Astragalus, Processed Aconite, Dried Ginger	(73)
Aikeqing Capsules	Yes	2017	536	268	3 pills each time, 3 times daily	CD4+	-	-	Epimedium, Ligustrum, Baical skullcap, Salvia, Polygonum, Licorice, Astragalus, Processed Aconite, Dried Ginger	(74)
Aikeqing Capsules	-	2018	132	-	3 pills each time, 3 times daily	CD4+	-	-	Epimedium, Ligustrum, Baical skullcap, Salvia, Polygonum, Licorice, Astragalus, Processed Aconite, Dried Ginger	(75)
Fuzheng Detox Tablets	-	2011	100	60	5 tablets, 3 times daily	CD4+	-	-	Coptis, Scutellaria, Astragalus, Angelica, Licorice, Ginseng, Atractylodes, Glossy Privet Fruit	(76)
Fuzheng Detox Tablets	-	2006	65	65	5 tablets, 3 times daily	CD4+	-	-	Coptis, Scutellaria, Astragalus, Angelica, Licorice, Ginseng, Atractylodes, Glossy Privet Fruit	(77)
Fuzheng Detox Tablets	-	2007	70	69	5 tablets, 3 times daily	CD4+, CD8+, CD4/CD8	Stabilized patient viral load, reduced partial patient viral load, 91.30% efficacy	Fuzheng Detox Tablets can block certain steps in the viral replication process to directly kill or clear the virus. It can help strengthen the body's resistance, promote specific and non-specific immunity, and thereby inhibit viral replication	Coptis, Scutellaria, Astragalus, Angelica, Licorice, Ginseng, Atractylodes, Glossy Privet Fruit	(78)
Fuzheng Detox Tablets	-	2016	110	110	5 tablets, 3 times daily	CD4+	64.1% efficacy	Combination of tonic and heat-clearing and detoxifying medicines, mainly tonics, to both boost the body's energy and attack pathogens and toxins	Coptis, Scutellaria, Astragalus, Angelica, Licorice, Ginseng, Atractylodes, Glossy Privet Fruit	(79)
Fuzheng Detox Tablets	-	2012	54	32	5 tablets, 3 times daily	-	Acts by regulating the interaction between CD40 and CD40L, thereby upregulating the expression of IFN-伪, enhancing its antiviral and immune regulation functions	-	Coptis, Scutellaria, Astragalus, Angelica, Licorice, Ginseng, Atractylodes, Glossy Privet Fruit	(80)
Ai Ling Granules	Yes	2005	60	60	2 packets, 2 times daily	CD4+, CD8+, CD4/CD8, CD4+CD45RA	Viral load remains stable, does not affect HARRT's ability to inhibit viral replication	-	Astragalus, Salvia, Peach Kernel	(81)
Ai Ling Granules	-	2008	17	17	2 packets, 2 times daily	CD4+	-	-	Astragalus, Salvia, Peach Kernel	(82)
Ai Ling Granules	-	2008	17	17	2 packets, 2 times daily	CD4+	-	-	Astragalus, Salvia, Peach Kernel	(83)
Ai Ling Granules	-	2006	19	19	2 packets, 2 times daily	CD4+	-	-	Astragalus, Salvia, Peach Kernel	(84)
Ai Ling Granules	-	2011	15	15	2 packets, 2 times daily	CD4+, CD45+RA	-	-	Astragalus, Salvia, Peach Kernel	(85)
Ai Ling Granules	-	2006	107	107	2 packets, 2 times daily	CD3, CD8, CD4, CD4/CD8	Viral load is stable or slightly reduced, but not statistically significant	-	Astragalus, Salvia, Peach Kernel	(86)
Ai Ling Granules	-	2006	21	21	2 packets, 2 times daily	CD3, CD8, CD4	Suppresses viral load to some extent or keeps it stable	-	Astragalus, Salvia, Peach Kernel	(87)
Ai Ling Granules	-	2009	21	19	2 packets, 2 times daily	CD3, CD8, CD4, CD4/CD8	Viral load remains stable	Ai Ling Granules can improve the patient's immune function and effectively control the rise of viral load, preventing rapid viral replication, keeping the patient in a non-disease stage, and maintaining stable conditions. The medicine is safe and has no obvious toxic side effects	Astragalus, Salvia, Peach Kernel	(88)
Ai Ling Granules	-	2010	45	45	2 packets, 2 times daily	CD3, CD8, CD4	-	-	Astragalus, Salvia, Peach Kernel	(89)
Ai Ling Granules	Yes	2007	60	30	2 packets, 2 times daily	CD3, CD4, CD8, CD4/CD8, CD4^*^CD45RA	No significant difference, has an inhibitory effect	-	Astragalus, Salvia, Peach Kernel	(90)
Ailing Granules	Yes	2012	58	40	2 packs each time, twice daily	CD3, CD4, CD8, CD4/CD8, CD4^*^CD45RA			Astragalus, Black Ginseng, Peach Kernel	(91)
Aikang Capsule	-	2015	86	43	5 capsules each time, 3 times daily				American Ginseng, Chinese Yam, Astragalus, Poria, Atractylodes, Rehmannia, Angelica, Donkey-hide Gelatin, White Peony, Ophiopogon, Goldthread, Licorice	(92)
Aikang Capsule	-	2008	102	65	5 capsules each time, 3 times daily	CD4+ and CD8+	-	-	American Ginseng, Chinese Yam, Astragalus, Poria, Atractylodes, Rehmannia, Angelica, Donkey-hide Gelatin, White Peony, Ophiopogon, Goldthread, Licorice	(93)
Aikang Capsule	-	2007	102	65	5 capsules each time, 3 times daily	-	-	-	American Ginseng, Chinese Yam, Astragalus, Poria, Atractylodes, Rehmannia, Angelica, Donkey-hide Gelatin, White Peony, Ophiopogon, Goldthread, Licorice	(94)
Kang Ai Bao Sheng Pills	-	2021	530	402	1 bag each time, 4 times daily	CD4+	-	Tonify Qi and nourish yin, clear heat and detoxify	Ginseng, Viola, Scutellaria, Atractylodes, Mulberry Bark, Ligustrum, Licorice	(95)
Kang Ai Bao Sheng Pills	-	2021	535	423	1 bag each time, 4 times daily	CD4+	Significant reduction in viral load	-	Ginseng, Viola, Scutellaria, Atractylodes, Mulberry Bark, Ligustrum, Licorice	(96)
Kang Ai Bao Sheng Pills	Yes	2017	300	150	1 bag each time, 4 times daily	CD3, CD8, CD4, CD45RA+, CD45RO+	Viral load remains stable	-	Ginseng, Viola, Scutellaria, Atractylodes, Mulberry Bark, Ligustrum, Licorice	(97)
Kang Ai Bao Sheng Pills	Yes	2017	126	63	1 bag each time, 4 times daily	CD4, CD3, CD8, CD28+, CD38+, CD45RA+, CD45RO+	HIV-1 viral load effectively suppressed	Improves patients' immune reconstruction	Ginseng, Viola, Scutellaria, Atractylodes, Mulberry Bark, Ligustrum, Licorice	(98)
Kang Ai Bao Sheng Pills	Yes	2012	84	84	1 bag each time, 4 times daily	CD4+	-	-	Ginseng, Viola, Scutellaria, Atractylodes, Mulberry Bark, Ligustrum, Licorice	(99)
Kang Ai Bao Sheng Pills	Yes	2012	179	179	1 bag each time, 4 times daily	CD4+	Tonify Qi and nourish yin, strengthen kidney and spleen, clear heat and detoxify	-	Ginseng, Viola, Scutellaria, Atractylodes, Mulberry Bark, Ligustrum, Licorice	(100)
Kang Ai Bao Sheng Pills	Yes	2023	71	35	1 bag each time, 4 times daily	CD3+, CD4+, CD8+	Viral load remains stable	Effectively improves the rheology of AIDS patients' blood, levels of inflammatory factors, enhances immune function, and has high safety	Ginseng, Viola, Scutellaria, Atractylodes, Mulberry Bark, Ligustrum, Licorice	(101)
Kang Ai Bao Sheng Pills	-	2011	18	18	1 bag each time, 4 times daily	CD4+	Detoxify and clear heat, activate blood and dispel dampness, nourish yin and benefit qi	-	Ginseng, Viola, Scutellaria, Atractylodes, Mulberry Bark, Ligustrum, Licorice	(102)
Kang Ai Bao Sheng Pills	-	2017	132	88	1 bag each time, 4 times daily	CD4+	Has a significant inhibitory effect	Emphasizes the liver, spleen, and kidney, multi-target and holistic regulation of immune function	Ginseng, Viola, Scutellaria, Atractylodes, Mulberry Bark, Ligustrum, Licorice	(103)
Kang Ai Bao Sheng Pills	Yes	2020	98	98	1 bag each time, 4 times daily	CD4+, CD8+	Detoxify and clear heat, activate blood and dispel dampness, nourish yin and benefit qi	-	Ginseng, Viola, Scutellaria, Atractylodes, Mulberry Bark, Ligustrum, Licorice	(104)
Kang Ai Bao Sheng Pills	-	2012	60	60	1 bag each time, 4 times daily	CD4+	No significant change in viral load		Ginseng, Viola, Scutellaria, Atractylodes, Mulberry Bark, Ligustrum, Licorice	(105)
Kang Ai Bao Sheng Pills	-	2015	60	60	1 bag each time, 4 times a day	CD4+		Ginseng, purple coneflower, scutellaria, atractylodes, mulberry bark, glossy privet fruit, licorice	Ginseng, Viola, Scutellaria, Atractylodes, Mulberry Bark, Ligustrum, Licorice	(106)
Ai Fu Kang Capsules	-	2012	198	132	4 pills each time, 3 times a day	CD4+	No significant change in viral load	Clear heat and detoxify, strengthen the spleen and warm the kidney, activate blood circulation and relieve pain	Scutellaria, honeysuckle, thunder god vine, Radix zanthoxyli, fraxinus bark, knotweed, polygonum cuspidatum alcohol, angelica, licorice	(107)
Ai Fu Kang Capsules	Yes	2017	50	25	4 pills each time, 3 times a day	CD4+	Both the treatment group and the control group had reduced viral loads after treatment (P > 0.05), no statistical significance between the two groups	Clear heat and detoxify, activate blood circulation and relieve pain	Scutellaria, honeysuckle, thunder god vine, both sides needle, fraxinus bark, knotweed, polygonum cuspidatum alcohol, angelica, licorice	(108)
Ai Fu Kang Capsules	Yes	2018	50	25	4 pills each time, 3 times a day	CD4+	Significant differences within the group, but no differences between groups	The baicalin and resveratrol in the drug may improve the patient's immune function	Scutellaria, honeysuckle, thunder god vine, both sides needle, fraxinus bark, knotweed, polygonum cuspidatum alcohol, angelica, licorice	(109)
Ai Fu Kang Capsules	Yes	2021	90	45	4 pills each time, 3 times a day	CD4+, CD8+	Virus suppression rate was 62.22%	Activate blood circulation and relieve pain, clear heat and detoxify, inhibit HIV, and enhance body immunity	Scutellaria, honeysuckle, thunder god vine, both sides needle, fraxinus bark, knotweed, polygonum cuspidatum alcohol, angelica, licorice	(110)
Fu Zheng Anti-AIDS Formula	-	2009	16	16	1 bag each time, 3 times a day	-	-	-	Astragalus, codonopsis, turmeric, licorice, etc.	(111)
Fu Zheng Anti-AIDS Formula	-	2007	30	30	1 bag each time, 3 times a day	CD4+, CD8+	The overall effective rate is 77.7%	-	Astragalus, codonopsis, turmeric, licorice, etc.	(112)
Fu Zheng Anti-AIDS Formula	-	2008	30	30	1 bag each time, 3 times a day	CD4+	The overall effective rate is 77.7%	-	Astragalus, codonopsis, turmeric, licorice, etc.	(113)
Tai Qi Pei Yuan Granules	Yes	2017	43	20	3 bags each time, 2 times a day	CD+3T, CD+8T, CD+4T	Increased, statistically significant	Tonify qi and nourish yin, nourish lung and kidney	Prince ginseng, rehmannia, ophiopogon, schisandra, astragalus, asparagus, angelica, goji berry, glossy privet fruit, yam, roasted atractylodes	(114)
Tai Qi Pei Yuan Granules	Yes	2022	60	30	3 bags each time, 2 times a day	Same as above	The difference is not obvious	-	Prince ginseng, rehmannia, ophiopogon, schisandra, astragalus, asparagus, angelica, goji berry, glossy privet fruit, yam, roasted atractylodes	(115)
Tai Qi Pei Yuan Granules	Yes	2022	38	18	3 bags each time, 2 times a day	Same as above	After treatment, there was no significant difference between groups	Improve the immune function of HIV-infected/AIDS patients	Prince ginseng, rehmannia, ophiopogon, schisandra, astragalus, asparagus, angelica, goji berry, glossy privet fruit, yam, roasted atractylodes	(116)
Tai Qi Pei Yuan Granules	Yes	2016	100	50	3 bags each time, 2 times a day	CD4+	-	Nourish yin and tonify qi, nourish kidney and moisten lung	Prince ginseng, rehmannia, ophiopogon, schisandra, astragalus, asparagus, angelica, goji berry, glossy privet fruit, yam, roasted atractylodes	(117)
Tai Qi Pei Yuan Granules	-	2017	108	54	3 bags each time, 2 times a day	CD4+, CD8+, CD4+/CD8+, CD3+	There was no statistically significant difference in the change of HIV-RNA before and after treatment (P > 0.05), indicating that Tai Qi Pei Yuan Granules had no significant effect on viral load	Tai Qi Pei Yuan granules have a good effect on improving the TCM symptoms of HIV-infected patients with qi and yin deficiency, lung and kidney insufficiency	Prince ginseng, rehmannia, ophiopogon, schisandra, astragalus, asparagus, angelica, goji berry, glossy privet fruit, yam, roasted atractylodes	(118)
Tai Qi Pei Yuan Granules	-	2017	81		3 bags each time, 2 times a day	CD4+ and CD8+	The difference is not obvious	-	Prince ginseng, rehmannia, ophiopogon, schisandra, astragalus, asparagus, angelica, goji berry, glossy privet fruit, yam, roasted atractylodes	(119)
Tai Qi Pei Yuan Granules	-	2016	54	27	3 bags each time, 2 times a day	-	-	-	Prince ginseng, rehmannia, ophiopogon, schisandra, astragalus, asparagus, angelica, goji berry, glossy privet fruit, yam, roasted atractylodes	(120)
Taiqi Peiyuan Granules	Yes	2020	88	44	3 bags each time, twice a day	CD4+, CD8+, CD4+CD45RA+	Can improve immune reconstitution efficacy	-	Prince ginseng, rehmannia, ophiopogon, schisandra, astragalus, asparagus, angelica, goji berry, glossy privet fruit, yam, roasted atractylodes	(121)
Taiqi Peiyuan Granules	Yes	2017	40	20	3 bags each time, twice a day	CD+3T, CD+8T, CD+4T	Increase, statistically significant	-	Prince ginseng, rehmannia, ophiopogon, schisandra, astragalus, asparagus, angelica, goji berry, glossy privet fruit, yam, roasted atractylodes	(122)
Immunity No.1	-	2012	72	36	1 bag each time, twice a day	CD4, CD45RA, CD45RO	-	-	American ginseng, astragalus, viola mandshurica, caterpillar fungus	(123)
Immunity No.1	Yes	2011	187	287	1 bag each time, twice a day	CD3/CD8/CD45/CD4 and others	No significant difference	-	American ginseng, astragalus, viola mandshurica, caterpillar fungus	(124)
Immunity No.2	Yes	2012	233	116	1 bag each time, twice a day	CD4+	Enhance qi, nourish yin, and replenish vitality	-	Astragalus, codonopsis, ganoderma, goji berry, horny goat weed, morinda, turmeric, American ginseng, caterpillar fungus, viola mandshurica, schisandra	(125)
Immunity No.2	Yes	2012	264	131	1 bag each time, twice a day	CD4+, CD45RO+ and CD45RA+	No significant difference	-	Astragalus, codonopsis, ganoderma, goji berry, horny goat weed, morinda, turmeric, American ginseng, caterpillar fungus, viola mandshurica, schisandra	(126)
Immunity No.2	Yes	2017	361	189	1 bag each time, twice a day	CD4+, CD45RO+ and CD45RA+	-	-	Astragalus, codonopsis, ganoderma, goji berry, horny goat weed, morinda, turmeric, American ginseng, caterpillar fungus, viola mandshurica, schisandra	(127)
Immunity No.2	-	2022	361	189	1 bag each time, twice a day	CD3+, CD4+, CD8+ and CD45RA+	Yes	-	Astragalus, codonopsis, ganoderma, goji berry, horny goat weed, morinda, turmeric, American ginseng, caterpillar fungus, viola mandshurica, schisandra	(128)
Immunity No.2	Yes	2013	264	131	1 bag each time, twice a day	CD4+, CD45RO+ and CD45RA+	Enhance qi, nourish lung, strengthen spleen and kidney	-	Astragalus, codonopsis, ganoderma, goji berry, horny goat weed, morinda, turmeric, American ginseng, caterpillar fungus, viola mandshurica, schisandra	(129)
Xiao Yao San combined with Er Chen Tang	-	2021	2021	80	1 bag each time, twice a day	CD4+ and CD8+	-	-	Bupleurum, angelica, atractylodes, salvia, hawthorn, alisma, pinellia, coix seed	(130)
Qian Kun Ning	-	2000	8	8	4 pills each time, three times a day	CD4+ and CD8+	Significant difference	-	Astragalus, polygonatum, scrophularia, poria, gardenia, coptis, citrus aurantium, forsythia, corydalis, houttuynia, turmeric, citrus aurantium, arisaema, galla rhois	(131)
Qian Kun Ning	-	2004	60	60	6 pills each time, three times a day	CD4+ and CD8+	Yes	-	Gardenia, caper spurge, forsythia, coptis, astragalus, polygonatum, poria, scrophularia, turmeric	(132)
Zhen Qi Fuzheng Capsules	Yes	2014	70	35	1 bag each time, twice a day	CD4+	-	-	Astragalus, glossy privet fruit	(133)
Zhen Qi Fuzheng Capsules	Yes	2017	129	59	1 bag each time, twice a day	CD4+	Enhance immune function; protect bone marrow and adrenal cortical function	-	Astragalus, glossy privet fruit	(134)
CKBM-A01	Yes	2009	18	-	“1 sachet each time, 2 times daily”	CD4+	No significant difference	-	Ginsenosides, Schisandra, Jujube powder, Hawthorn, Green beans, Glycine, Brewing yeast, Apple, Honey, Water	(135)
IGM-1	Yes	1996	30	15	“4 times daily, 7 tablets each time”	CD4+	-	-	Ganoderma, Isatis, Astragalus membranaceus, Andrographis paniculata, Andrographis echioides, Peristrophe roxburghiana, Hedyotis diffusa, Kelp japonica	(136)
XQ-9302	-	1999	48	48	“6 pills each time, 4 times daily”	CD4+	-	Inhibits HIV replication within the human body	Rhubarb, Phellodendron, Goldthread, Kelp, Seaweed, Fresh oysters, Monkey dates	(137)
ZL-1 Capsule	-	2013	120	60	“4 sachets each time, 4 times daily”	CD4+	-	The provided mechanism describes a combination of ingredients that balance and harmonize, achieving both suppression of virus replication and enhanced immune function.	Poria cocos, Rubia cordifolia, Ginseng soap base, Caterpillar Fungus powder	(138)
Research No. 4	-	2006	63	30	“2 packets each time, 2 times daily”	CD4+, CD45RA +, and CD8 +	Better than placebo group	-	Ginkgo, Astragalus, Goji, Safflower, Scutellaria, Bletilla striata	(139)
Wenbu Pishen Granules	Yes	2012	40	20	“1 sachet each time, 2 times daily”	CD4+CD8+	-	Warm and nourish spleen and kidney, enhance qi and blood	Yam, Gastrodia, Rhynchophylla: sour Jujube Kernel, Yuanzhi, Acacia bark 3; Baikal Skullcap, Cypress, Wheat; Honeysuckle, Forsythia, XuanShen; meat, dog spine, Corydalis.	(140)
Wenshen Jianpi recipe	Yes	2022	56	28	“1 sachet each time, 2 times daily”	CD4+	-	-	Ginseng, Ganoderma, Atractylodes, Poria, Dodder, Morinda, Goji	(141)
Yang deficiency and dampness stagnation type	Yes	2021	66	32	“1 sachet each time, 3 times daily”	CD4+	“Below the detection limit (<100 copies/mL), stable viral suppression”	-	Cinnamon, vinegar tortoise shell, Amomum villosum, codonopsis, white atractylodes, Buddha's hand, Poria, epimedium, dry ginger, roasted licoric, Salvia miltiorrhiza, Angelica Sinensis, Sheng Long Bone, Lycium barbarum, Accumulator shells	(142)
Xiang A1 Granules	Yes	2022	42	21	2 times daily, 1 sachet each time	CD4+ T lymphocytes	-	Increase the number of CD4+ T lymphocytes in patients with HIV/AIDS with spleen deficiency and dampness, possibly by regulating the balance of Th1/Th2 cells	Coix lacryma, Chinese yam, Poria, Cardamomum, Patchouli, Acorus calamus; Rhizoma Pinelliae, Rhizoma Pinelliae, Forsythia, Cortex Eucommiae, Peppermint, Mouton, Slippery Rock, Licorice	(143)
New Century Kangbao Capsule	-	2000	43	43	“2 capsules daily, 2 times daily”	CD4+	-	-	Selenium-rich polysaccharides from seaweed and glycyrrhizic acid	(144)
Xin Xue Pian (Tang Grass Tablet)	Yes	2006	49	25	-	CD4+	“After 6 months of treatment, the virus inhibition degree of the control group was significantly better than that of the treatment group, with P <0.5”	-	Honeysuckle, Trichosanthes kirilowii peel, Bupleurum	(145)
Artemisinin Ester Tablet	Yes	2022	45	29	“50mg each time, 2 times daily”	CD4+	-	-	-	(146)
Qu Du Zeng Ning Capsule	-	2019	26	13	-	CD4+	88.46%	Clear heat and detoxify, nourish qi and yin, strengthen the middle	-	(147)
Shuang Huang Lian Powder Injection	-	1999	15	15	“60mg/kg, once daily IV drip”	CD3+CD4+CD8	-	Direct inhibitory effect on reverse transcriptase	Honeysuckle, Scutellaria, Forsythia	(148)
Pulekang Oral Solution	-	2005	12	12	100ml/each time, 3 times daily	CD4+CD8+	Decreased by 129.16cp/ml	-	-	(149)
Compound Astragalus Granules	-	2009	-	-	“3 times daily, 1 sachet each time”	CD4+CD8+	-	Increase CD4+ cell count in HIV infected individuals, certain inhibitory effect on HIV replication, and some immunoregulatory effects	Astragalus, Ginseng, Angelica, Goji Berry, Licorice	(150)
Compound Astragalus and Atractylodes Decoction	Yes	2014	90	30	“2 times daily, 1 packet each time”	CD4+	-	-	Astragalus, Codonopsis, Atractylodes macrocephala, Poria cocos, Ganoderma lucidum, Lithospermum, Fangfeng	(151)
Compound Three-Yellow Capsule	-	2011	48	32	-	CD4+CD8+	“Certain inhibitory effect on HIV, viral load decreases nearly 1 log copies/mL”	“Stimulate T cell proliferation in the infected, adjust T cell subpopulation disorder; Might enhance CD4+ T cell function, rectify immune dysfunction; Possibly enhance NK cell activity; Might have a direct inhibitory effect on HIV proliferation.”	Oldenlandia, Violet, Licorice, Astragalus, Phellodendron, Angelica, Bupleurum, Fangfeng, Hedyotis diffusa, Atractylodes, Dandelion, Dodder, Scutellaria, Zedoary	(152)
Tripterygium wilfordii Hook F. Multiglyco-side Pills	-	2022	33	-	-	-	Post-treatment HIV viral load decreases, the trend is described as viral load reduction	The mechanism of TPL is described as inhibiting immune cell activation, production of IFN-纬, expression of downstream interferon-stimulated genes and phosphorylation of STAT1	Root of Tripterygium wilfordii	(153)
Aining Granules	-	2012	43	43	“2 times daily, 20g each time”	CD4+ and CD8+	-	Enhance immune function, control opportunistic infections, improve quality of life, allow patients to live with the virus	-	(154)
“Shenling Fuzheng Capsules, Qingdu Capsules”	Yes	-	100	67	4 pills each time; 3 times daily	CD4+CD8+	-	-	Codonopsis, Astragalus, Baicalin, Ganoderma, Kudzu root and crude extract of resveratrol; Astragalus, Atractylodes, Scutellaria, Andrographis paniculata, Copper yeast, Wolfiporia extensa, Coix seed, Cardamom, Ganoderma, Poria cocos crude extract	(155)
Fuyang Detoxification Granules	-	2014	25	25	1 sachet each time, 2 times daily	CD4+	Reduces viral load	Warm and tonify yang, nourish qi and yin, detoxify and dispel dampness	Corydalis yanhusuo, Half Branch Lotus, Epimedium, Deer Antler, Polygonatum, Codonopsis, Bupleurum, Pinellia ternata, Poria	(156)

### 3.1 Yi Ai Kang capsules

Yi Ai Kang capsules contain an herbal formulation with a rich composition, including ginseng, *Astragalus, Ligusticum wallichii*, fried atractylodes, *Rehmannia, Ophiopogon, Poria cocos, Angelica sinensis*, peony, scutellaria, and licorice, endowing them with various pharmacological properties such as invigorating Qi, fortifying the spleen, nourishing Yin, dispelling wind and heat, and detoxifying dampness (157). Recently, numerous clinical studies have confirmed the potential efficacy of Yi Ai Kang capsules in treating patients with AIDS, particularly those with lung-spleen Qi deficiency syndrome (65–69). In addition to improving clinical symptoms and signs, they reduce the incidence of opportunistic infections, stabilize viral loads, increase CD4+ T lymphocyte counts, prolong patient survival, and lower mortality rates (62–70, 157, 158).

According to Li et al. (62), Yi Ai Kang capsules, in conjunction with traditional Chinese syndrome differentiation treatment, notably improved clinical symptoms and signs, reduced viral load, increased CD4+ T lymphocyte counts, and decreased the occurrence of opportunistic infections. Zhang (65) also employed a combination of Yi Ai Kang capsules and traditional Chinese syndrome differentiation treatment, suggesting that the mechanism of improving immune function in patients may involve blocking the PD-1 and ID2/E2AmRNA signaling pathways, thereby enhancing immune function. The clinical expert group of the Henan Provincial Traditional Chinese Medicine AIDS Treatment emphasizes that intervention with Yi Ai Kang capsules in patients with HIV can stabilize or slow the decline in CD4+ T lymphocytes, reduce viral loads, enhance quality of life, and extend survival (59). Yang (64) pointed out that combining Yi Ai Kang capsules with HAART can improve quality of life by alleviating clinical symptoms and increasing CD4+ T lymphocyte counts. Research by Wang (66) demonstrated that combining Yi Ai Kang capsules with HAART is more advantageous than HAART monotherapy for immune reconstitution in patients with poor immune function (lung-spleen Qi deficiency syndrome). Furthermore, Zhang et al.'s study (70) suggests that combining Yi Ai Kang capsules with a cough-relieving formula effectively improves cough symptoms in patients, achieving an overall efficacy rate of 93.75%. The Yi Ai Kang capsules also exhibit the potential to regulate immune functions. According to research by Liu et al. (67), the Yi Ai Kang capsules promote a balanced ratio of Th17/Treg cells, effectively regulate the disruption of the gut microbiota, and enhance immunity. Li et al. (68) found that Yi Ai Kang can improve the expression of helper receptors and abnormal cell activation levels in patients with AIDS, thereby mitigating immune damage following HIV infection. Zhao et al. (159) demonstrated the regulation of immune function by a combined treatment of Yi Ai Kang capsules combined with IPVr, promoting the differentiation of CD4+ T cells into CD4Tcm cells. Additionally, Xu (69) revealed that Yi Ai Kang capsules can reduce viral replication and immune damage by modulating the NF-κB signaling pathway.

### 3.2 Aikeqing capsules

Aikeqing capsules, a TCM herbal compound, contain various medicinal herbs, including *P. vulgaris, Scutellaria baicalensis, S. miltiorrhiza, Epimedium, P. cuspidatum, Astragalus membranaceus, Glycyrrhiza uralensis, Eucommia ulmoides, Lithospermum erythrorhizon*, and *Atractylodes macrocephala*. These capsules possess the therapeutic properties of heat clearing, detoxification, kidney tonification, Qi enhancement, improving blood circulation, and stasis removal. Aikeqing capsules have various applications in the treatment of patients with HIV/AIDS who have spleen and kidney deficiencies, as well as lingering pathogenic factors, to ameliorate clinical symptoms and enhance the quality of life. With advances in clinical research, numerous studies have confirmed the therapeutic efficacy of Aikeqing capsules in the treatment of patients with HIV/AIDS. This paper provides a brief overview and analysis of the representative studies to serve as a reference for exploring the efficacy of Aikeqing capsules in HIV treatment.

The effectiveness of Aikeqing capsules for the treatment of HIV/AIDS has been validated in multiple clinical studies. Li and Zuo (71, 72) demonstrated that prolonged use of Aikeqing capsules alone can stabilize and elevate CD4+ T lymphocyte counts in patients with HIV, especially for those with CD4+ T counts in the range of 200 to 350 cells/μL. Cao and Dong et al. (73, 74) combined Aikeqing capsules with HAART treatment for over 12 months and demonstrated improved CD4+ T lymphocyte counts and ameliorated clinical symptoms compared with those of HAART monotherapy. Furthermore, Wang et al. examined the effect of Aikeqing capsules on post-HAART inflammation in patients with HIV/AIDS from the perspective of ADAM17. Although there were no significant differences in certain indicators, this study highlights the regulatory role of Aikeqing capsules (75). In summary, Aikeqing capsules demonstrated potential efficacy in the treatment of patients with HIV/AIDS. They can stabilize and elevate CD4+ T lymphocyte counts, ameliorate clinical symptoms, enhance quality of life, and show a certain level of safety for use. Further large-scale clinical trials are required to validate the precise efficacy and mechanism of action of Aikeqing Capsules for the treatment of HIV/AIDS.

### 3.3 Fuzheng Paidu pills

Fuzheng Paidu pills are compound Chinese herbal medicines containing ingredients such as *A. membranaceus, Panax notoginseng, Ligustrum lucidum, Cornus officinalis, Hedyotis diffusa, Forsythia suspensa, A. macrocephala, Saposhnikovia divaricata*, and *G. uralensis* (76). They have properties of replenishing Qi and nourishing Yin, clearing heat, and causing detoxification. They have demonstrated significant efficacy in clinical applications and are available as mature formulations. Fuzheng Paidu pills improve symptoms and signs of “deficiency of righteous Qi and lurking pathogenic factors” in asymptomatic individuals infected with HIV (160). They increase or stabilize CD4 cell counts in infected individuals and reduce the viral load. This medication improves the overall quality of life of infected individuals and has been safely administered in clinical practice. Furthermore, Fuzheng Paidu pills regulate the human immune system, enhance body resistance, and alleviate patient discomfort.

Peng and Wang (77) found that Fuzheng Paidu Pills maintain the stability of CD4+ T cell counts in patients with an effective rate of 82.69%, significantly improving clinical symptoms and enhancing immune function. Similarly, Liu (78) demonstrated a decrease in major symptoms of a patient to varying degrees after treatment with Fuzheng Paidu pills, with a treatment effectiveness rate of 91.3% for viral load reduction. Multiple studies have revealed the mechanisms of action of Fuzheng Paidu pills in HIV/AIDS treatment. According to Jiang et al. (80), Fuzheng Paidu pills upregulate IFN-α expression by regulating the interaction between CD40 and CD40L, thereby enhancing antiviral and immune-regulatory functions. Additionally, Peng et al. (161) demonstrated that Fuzheng Paidu pills enhanced the phagocytic function of peritoneal macrophages in immunosuppressed mice, promoted the formation of hemolysin, and increased the transformation of peripheral blood lymphocytes. Tan suggested that Fuzheng Paidu pills increase CD4 + T lymphocyte counts, stabilize viral load, and protect liver function when used to treat HIV/HCV co-infection (79). Shi et al. (162) further revealed that these pills significantly increase CD3+ and CD4+ cell counts in immunosuppressed mice, elevate the CD4+/CD8+ ratio, and improve overall immune function. In summary, Fuzheng Paidu pills have exhibited significant efficacy in the treatment of HIV/AIDS. They increase or stabilize CD4+ cell counts, ameliorate clinical symptoms, enhance quality of life, and possess a certain degree of immunoregulatory and antiviral activity.

### 3.4 Ailing Granules

Ailing Granules, a traditional Chinese herbal compound, mainly consist of ingredients such as *A. membranaceus, S. baicalensis*, and *Persica*. These granules possess the medicinal properties of primarily targeting symptoms of Qi deficiency and blood stasis while also tonifying Qi, nourishing Yin, promoting blood circulation, and detoxifying (81, 88, 90). They are used in the clinical treatment of patients with HIV/AIDS who exhibit a combination of deficiencies and excess syndromes, providing a holistic approach to restoring health. This article provides a comprehensive analysis of multiple clinical studies to explore the composition, therapeutic effects, specific indications, and clinical treatment outcomes of Ailing Granules, providing a reference for the application of traditional Chinese medicine in HIV/AIDS treatment.

Several clinical studies have explored the application and therapeutic effects of Ailing Granules in HIV/AIDS treatment. In a study by Liu (81), Ailing Granules, when used in conjunction with HAART for long-term treatment of patients with HIV/AIDS who had Qi deficiency and blood stasis, improved clinical symptoms and signs, increased CD4+ T lymphocyte counts, enhanced immune function, and did not compromise the effectiveness of the antiretroviral therapy. Multiple studies conducted by Jian'an also demonstrated that Ailing Granules control the increase in viral load (82, 84, 86), inhibit HIV-1 virus replication (86, 87), increase CD4+ T lymphocyte counts (82, 85, 87), improve patient symptoms and signs (84), enhance immune function (83, 84, 87), and stabilize HIV-infected individuals (82–88). In research focusing on patients with HIV/AIDS who have Qi deficiency, blood stasis, and lingering pathogenic factors, Ailing Granules also exhibited significant therapeutic effects (88). According to Song et al. (89), Ailing Granules might influence the progression of AIDS by restoring CD4+ T cell counts, enhancing their IFN-γ secretion capacity, and reducing CD8+ T cell secretion of IL-4, thereby rebalancing Th1 and Th2 cytokines. Jin (90) indicated that Ailing Granules, in combination with HAART, promoted the secretion of IFN-α by MDC and PDC, thereby improving patient immune function. This finding was also supported by other studies (81–83). According to Chen et al. (91), in individuals infected with HIV, Ailing Granules could, to some extent, delay the shift of Th1-type cells to Th2-type cells, rebalancing Th1 and Th2 cytokines. According to multiple studies, Ailing Granules increase CD4+ T cell counts, enhance immune function, and stabilize the viral load. Some studies have focused on patients with Qi deficiency and blood stasis, while others have included many types of patients with HIV/AIDS. These differences could have an impact on the specificity and generalizability of the results.

### 3.5 Kangai capsules

Kangai capsules comprise 21 TCM herbs, including *Viola yedoensis, S. baicalensis, Morus alba bark, P. vulgaris, Isatis indigotica, Panax ginseng, A. membranaceus, Dioscorea oppositifolia*, and *P. cocos*. These herbs contain multiple active ingredients and exhibit various pharmacological actions, including immune regulation, anti-inflammation, and antioxidation. Kangai capsules have been demonstrated in multiple studies to improve HIV/AIDS treatment primarily by increasing CD4+ T cell counts (95–106), controlling viral load (96, 103, 105), and enhancing immune function (95, 99, 101). Wang et al. (95) found that Kangai capsules significantly improved CD4+ T cell counts in patients, aiding in maintaining immune function. According to Li (97), Kangai capsules combined with HAART increased CD4+ T cell numbers, improved immune function, alleviated the extent of venous dilatation, and lowered the levels of inflammatory factors. These studies demonstrated the positive effects of Kangai capsules in improving the immune status of patients with HIV/AIDS. He et al. (101) observed that the combination therapy of HAART with Kangai capsules reduced plasma viscosity and improved inflammatory factor levels in patients. Clinical studies have shown that Kangai capsules do not exhibit significant peripheral blood cell toxicity (104) and may even protect kidney function (103). In clinical observations conducted by Ba (106), for HIV-infected individuals with inherent factors of dampness and Qi-Yin deficiency, Kangai capsules improved symptoms and signs at the T-cell level. Kangai capsules have exhibited a positive impact on CD4+ T cell counts, immune function, viral load, and quality of life in different studies (95, 96, 98, 100, 102). Furthermore, some studies have explored the application of Kangai capsules in different patient groups, such as intravenous drug users and those with sexually transmitted infections (99), providing novel directions for further research. In summary, Kangai capsules demonstrated significant efficacy and potential for HIV/AIDS treatment. Their mechanisms of action may involve immune regulation, anti-inflammation, and antioxidation, involving multiple pathways.

### 3.6 Aifukang capsules

Aifukang capsules, a TCM preparation, have shown unique therapeutic effects and potential for HIV/AIDS treatment. Aifukang capsules are composed of traditional Chinese herbs, including *S. baicalensis, Lonicera japonica, Clematis chinensis, Herba Lycopi, Cortex Fraxini*, and *P. cuspidatum*. These ingredients have functions such as clearing heat, detoxifying, promoting blood circulation, and removing obstructions in the meridians. Wu et al. (107) demonstrated that Aifukang capsules improve immune function of patients, increase body weight, and alleviate clinical symptoms such as fatigue, decreased appetite, and headache. This suggests that Aifukang capsules contain components that enhance immune system function and alleviate symptoms.

Aifukang capsules have shown a series of positive therapeutic effects in HIV/AIDS treatment. According to Mao et al. (108) and Mao (109), Aifukang capsules improved immune indicators and reduced the viral load in patients with HIV/AIDS with hot toxins and internal accumulation while alleviating the toxic side effects of HAART (110). However, owing to the differences between various studies, further clinical and basic research is needed to validate its efficacy and safety. In summary, the application prospects of Aifukang capsules in HIV/AIDS treatment remain promising, offering novel insights and approaches for the development of the field.

### 3.7 Fuzheng Kang'ai formula

The Fuzheng Kang'ai Formula is composed of various traditional Chinese herbs, including *A. membranaceus, Codonopsis pilosula, Curcuma longa*, and *G. uralensis*. This formula strictly adheres to the treatment principle of “strengthening the body's foundation to eliminate the root cause while dispelling pathogenic factors to address the symptoms.” It combines TCM knowledge with modern pharmacological research to create well-balanced formulations. This formula balances nourishment and purging with cold and warm properties, promotes blood circulation to dispel toxins, and clears blockages to help the body resist and expel pathogens (111). It is suitable for patients with AIDS exhibiting symptoms of “Qi deficiency and blood stasis.”

Liu (112) showed that Fuzheng Kang'ai Formula significantly improved patient symptoms and signs, increased CD4 cell levels, inhibited and stabilized viral replication, and had some inhibitory effects on CCR5 and CXCR4 expression. Additionally, the formula effectively alleviated symptoms such as fatigue, poor appetite, hair loss, abdominal distension, and muscle pain in patients with AIDS diagnosed with the TCM pattern of “Qi deficiency and blood stasis” (113). Liu (163) further confirmed that the formula could enhance cellular immune function in mice and block HIV infection through competitive binding with CCR5. These findings suggest that the Fuzheng Kang'ai Formula contains components that enhance immune function, promote blood circulation to eliminate toxins, and inhibit viral replication.

The Fuzheng Kang'ai Formula has shown significant therapeutic effects in clinical studies. Multiple studies have shown that this formula enhances immune function, alleviates clinical symptoms, stabilizes viral load, and slows disease progression. Wang (111) demonstrated that the Fuzheng Kang'ai Formula increased the secretion of IL-2 and IFN-γ, elevated T cell counts and enhanced immune function. The Fuzheng Kang'ai Formula demonstrated enhanced treatment effects when used in conjunction with antiretroviral drugs (HAART). The effect of the formula on T cell surface PD-1 expression and CD8+ T lymphocyte count varies among different studies. Some studies have also explored the mechanism of action of the Fuzheng Kang'ai Formula, such as its regulation of phospholipid transport and its effects on the TLR4 receptor. Clinical observations have shown that the Fuzheng Kang'ai Formula is safe for treating patients with HIV/AIDS, which synergistically enhances immune reconstruction and improves quality of life, especially when used in combination with HAART.

### 3.8 Taiqi Peiyuan granules

Taiqi Peiyuan granules are a TCM compound preparation composed of various Chinese herbs. The major ingredients include *A. membranaceus, C. pilosula, C. longa, Rehmannia glutinosa, Ophiopogon japonicus*, and *G. uralensis*. These herbs are primarily used for patients with HIV/AIDS who exhibit deficiencies in both Qi and Yin and lung and kidney deficiencies. Multiple clinical observational studies have shown that Taiqi Peiyuan granules improve immune function (114, 115, 121), increase CD4+ T lymphocyte counts (114–116, 118), raise the CD4+/CD8+ ratio, enhance immune function, control virus replication (114–116), and maintain the health of patients (114–117, 120–122, 164). The formula also has a positive impact on clinical symptoms in patients with HIV/AIDS, presenting with symptoms such as insomnia, restlessness, night sweats, spontaneous sweating, fatigue, susceptibility to cold, and phlegm production (116). Taiqi Peiyuan granules influence the expression of PD-1 on T cell surfaces, affecting T cell activation (115). The formula assists in reducing the viral load when used in combination with antiretroviral drugs, possibly owing to its regulation of immune cell function (116). Clinical observations have indicated that Taiqi Peiyuan granules are safe for the treatment of HIV/AIDS, particularly when used in conjunction with HAART, as this combination can synergistically enhance immune reconstitution and improve quality of life of patients (114, 115, 117–119, 121).

### 3.9 Immunity 1

Immunity 1 primarily consists of ingredients such as western ginseng, *Cordyceps sinensis*, and *Isodon amethystoides*. It is a TCM compound granule developed based on the mechanism of HIV/AIDS through long-term clinical practice for the prevention and treatment of AIDS, with efficacy in strengthening the body and providing detoxification. Immunity 1 effectively improved immune function in patients with latent HIV/AIDS. According to research findings, immunity 1 mainly acts on the elevation of CD45RO; however, after 6 months of treatment, it mainly acts on CD45RA (123). Combining Immunity 1 with antiretroviral therapy (HAART) significantly promoted immune reconstitution in patients with HIV/AIDS. Immunity 1 treatment effectively improves symptoms and signs in patients with HIV/AIDS, particularly digestive system symptoms, such as poor appetite and diarrhea (124). As a TCM prescription, Immunity 1 exhibits a significant immune regulatory effect in the treatment of patients with AIDS. By promoting improved immune function, Immunity 1 helps to elevate the level of immune reconstitution in patients, especially those with HIV/AIDS.

### 3.10 Immunity 2

Immunity 2 is a TCM compound prescription that mainly includes Western *ginseng, C. sinensis* mycelia, and *Schisandra chinensis*, among other ingredients. By adhering to the principles of tonifying the body and eliminating pathogenic factors while clearing heat and detoxifying, Immunity 2 aims to strengthen the body's foundation, resist pathogenic factors, promote immune reconstitution, and either suppress disease progression or maintain a balance between health and disease in patients with AIDS. Immunity 2 increases the absolute count of CD4 cells in patients, promotes immune reconstitution, and effectively improves immune function (125, 129). Treatment with Immunity 2 significantly improves symptoms such as fatigue, joint and muscle pain, skin itching, and shortness of breath, and enhances the quality of life (126, 165). Studies have indicated that Immunity 2 can partially restore CD45RA and CD45RO cell subpopulations (129). Additionally, when treating patients with HIV/AIDS with immune reconstitution deficiency, Immunity 2 affects the TLR9-mediated signaling pathway, which may be one of the mechanisms for improving abnormal immune activation (165). Immunity 2 treatment can increase the CD4 lymphocyte count in patients with immune reconstitution deficiency, thereby enhancing the body's immune response (127). Immunity 2 demonstrates significant therapeutic effects in patients with immune reconstitution deficiency. Through immune function regulation and the promotion of increased CD4 cell counts, this prescription effectively improves clinical symptoms, enhances quality of life, and helps restore abnormal immune states.

### 3.11 Other traditional Chinese medicine compound formulations

Several clinical trials have shown that various other TCM preparations had positive effects on the immune function and clinical symptom improvement of patients with HIV/AIDS (130, 132, 156). These TCM formulations help in increasing CD4+ T lymphocyte counts, reducing the viral load, improving immune function, alleviating clinical symptoms, and ultimately enhancing the quality of life of patients. For example, Qian Kun Ning, Zhen Qi Fu Zheng capsules, and compound *Astragalus* granules have been shown to significantly increase CD4+ T cell counts, thereby enhancing immune function (132, 133, 138, 148, 150). In addition, Xiao Yao San He Er Chen Tang, Wen Bu Pi Shen Fang, Pu Le Kang Oral Liquid, and other TCM formulations have been effective in inhibiting virus replication, reducing viral load, improving clinical symptoms, and significantly enhancing quality of life of patients (130, 133, 137, 139, 140, 154). Additionally, improvements in clinical symptoms, such as increased body weight and reduced fatigue, have been observed with the use of these formulations (137, 142, 151). However, discrepancies were there in the results, with some studies not observing significant therapeutic effects (135, 150), which may be attributed to factors such as the specific formulation and the study population. Safety assessment is another crucial aspect of these studies, with most indicating that TCM formulations are safe for treatment with no apparent adverse reactions or toxic side effects (133, 152, 154). However, some studies reported potential side effects that require further verification (146, 153). This underscores the importance of a high level of safety in TCM treatment.

Nonetheless, different TCM formulations exhibit differences in their treatment efficacy and mechanisms. Clinical observational data indicate that the efficacies and mechanisms of action of various TCM formulations are not identical. For example, Qian Kun Ning primarily improves patients' blood lipid levels and clinical symptoms by alleviating symptoms related to liver depression, spleen deficiency, and phlegm stagnation (131). Conversely, Qian Kun Ning effectively reduces mortality and plasma viral load and increases CD4+ T cell counts in patients with HIV/AIDS (132). Zhen Qi Fu Zheng capsules, when used in combination with HAART, increase neutrophil and CD4+ T lymphocyte counts, improve quality of life, and reduce adverse reactions to HAART drugs (133). Therefore, while TCM treatment shows positive effects in most cases, decisions regarding treatment options should consider the specific circumstances of the patients and be supported by data from clinical trials.

## 4 Pharmacological experimental results of traditional Chinese medicine compound formulations

Compound herbal formulations have been extensively examined through *in vitro* and *in vivo* animal studies to understand their mechanisms of action against HIV. These studies investigated their impact on aspects such as viral replication, viral invasion of host cells, and immune regulation. Various active ingredients within compound formulations may exert their effects through different pathways, including the inhibition of HIV viral replication enzymes, anti-inflammatory actions, and modulation of cytokine production. Some pharmacological experiments have shown that compound formulations inhibit HIV replication, reduce viral load, and modulate the immune system (166). Additionally, some compound formulations promote the growth and functional recovery of CD4+ T lymphocytes (162, 163). Qian et al. (158) and Zeng et al. (164) found that Yi Ai Kang Capsules and Taiqi Peiyuan granules, respectively, inhibited NK cell apoptosis and regulated the immune function of immunocompromised mice by increasing NK cell numbers (158, 164). Yi Ai Kang Capsule belong to one kind of TCM which is composed of various herbs, including *Ginseng, Astragalus membranaceus, Saposhnikovia divaricate*, and others, with the function of alleviating the symptoms of AIDS opportunistic infection. Yi Ai Kang Capsules have been demonstrated to lower the permeability of the intestinal mucosal barrier damaged by HIV, reduce cell apoptosis rates, and maintain the integrity of the intestinal mucosal barrier (167–169). Yi Ai Kang Capsules can alleviate lopinavir-induced lipid metabolism disorders, promote the uptake and transport of fatty acids, and enhance lipid clearance, thereby exerting a lipid-lowering effect (168). Fuzheng Paidu tablet includes Western *ginseng, Astragalus membranaceus, Forsythia suspensa* and other traditional Chinese herbs. Fuzheng Paidu tablet regulates the immune function of immunosuppressed mice, primarily by promoting lymphocyte transformation and enhancing the formation of hemolysins and hemolysis plaques (161, 162).

## 5 Compound herbal formulations combined with HAART treatment: a novel therapeutic strategy

In clinical studies, TCM preparations are frequently used as adjunctive therapies, often combined with HAART, to improve treatment outcomes. Different TCM preparations have demonstrated diverse synergistic effects when used in combination with HAART. For example, Zhen Qi Fu Zheng Capsules, one kind of oral TCM, mainly include *Astragalus membranaceus* and *Ligustrum lucidum*. Zhen Qi Fu Zheng Capsules combined with HAART reduce the adverse effects of HAART drugs and increase CD4+ T lymphocyte counts, and they have no significant impact on HIV viral load conversion rates (133). Similarly, when combined with HAART, Wen Bu Pi Shen Fang and Wen Shen Jian Pi Fang promote immune reconstitution in HAART non-responders, improve the distribution of NK cell subpopulations, and enhance patient quality of life (140, 141). This suggests that using TCM in conjunction with HAART will further enhance treatment effectiveness, improve immune function, and reduce treatment-related adverse reactions.

In clinical settings, compound herbal formulations are typically used in conjunction with modern antiretroviral therapy (HAART) to achieve better treatment outcomes. The role of herbal formulations in this combined treatment approach may involve several factors such as:

1. Synergistic viral suppression: Compound herbal formulations with HAART drugs synergistically inhibit viral replication via different pathways, thereby enhancing the effectiveness of viral replication inhibition.
2. Immune function restoration: Compound herbal formulations may help repair the damaged immune systems of patients with HIV/AIDS through immune modulation, increasing the body's natural defense mechanisms and strengthening its ability to combat the virus.
3. Mitigation of side effects: Some components within compound herbal formulations may possess anti-inflammatory and antioxidant properties, potentially helping to alleviate the side effects of HAART drugs and improve quality of life of patients.

The role of herbal formulations is characteristic by combining compound with HAART should be performed under the guidance of a medical professional, with dosages and treatment plans adjusted according to the specific circumstances of each patient. Further clinical trials and in-depth studies are needed to validate the safety and efficacy of this combined treatment strategy.

## 6 Conclusion and outlook

### 6.1 Advantage of the effects of TCM on HIV/AIDS treatment

Human immunodeficiency virus and AIDS are major global health problems. Despite significant advances in modern antiretroviral therapy (ART), issues related to long-term side effects, drug resistance, and other challenges persist. Herbal medicines have garnered significant attention from scientists as a potential adjunctive treatment. This study aimed to explore the role of herbal medicines in treating HIV/AIDS.

The role of herbs in HIV/AIDS treatment extends beyond direct antiviral activity; it also includes the modulation of the host immune system. Certain herbs enhance immune function and reduce immune suppression, thereby reducing the risks of infection and disease progression. Additionally, some TCM possess anti-inflammatory, antioxidant, and antifibrotic properties, which reduce the side effects of ART and improve the overall quality of life.

### 6.2 Limitation of TCM in HIV/AIDS treatment

Although herbal medicines have therapeutic potential in HIV/AIDS treatment, many unknown areas require further research. First, a deeper understanding of the specific antiviral mechanisms of herbs is required, including their effects at different stages of the viral life cycle. Current studies showed that several herbal medicine, such as *Golobe Halmahera, Pangium edule Reinw*, and Love herbal (LH) medicine, have significant antiviral effects (170–172). The components of these herbal medicine have the potential to be developed into chemotherapeutic drugs, especially with the clear acquisition of some of their structures, which allows for in-depth functional research. Therefore, to explore the structures of chemical compositions of TCM mentioned in this review is also a focus of future research.

Second, rational combinations of herbs enhance their therapeutic effects, and the synergistic interactions between herbal medicines require further exploration. Furthermore, optimizing the dosage forms of herbal preparations is crucial for ensuring the stability and bioavailability of active ingredients.

### 6.3 Future work on TCM in HIV/AIDS treatment T

In summary, herbal medicines show promise for the treatment of HIV/AIDS. In addition to directly inhibiting viral activity, they also regulate immune function and reduce adverse reactions. However, the therapeutic effects of TCM need further research and validation. Future studies should focus on the antiviral mechanisms of herbs, rational combinations, and dosage form optimization. In clinical applications, to ensure the best treatment outcomes for patients, caution should be exercised, and the guidance of modern medicine should be considered. Herbal medicine holds vast potential as an adjunctive treatment for HIV/AIDS and is expected to provide more treatment options and improve the quality of life of patients with HIV/AIDS. Therefore, clarifying the efficacy and mechanisms of TCM in treating HIV/AIDS and improving diagnosis and treatment system for AIDS would be the future research theme.

## Author contributions

NZ: Funding acquisition, Investigation, Writing – original draft. MW: Formal analysis, Validation, Writing – original draft. LG: Investigation, Supervision, Writing – original draft. CZ: Investigation, Writing – original draft. XT: Investigation, Writing – original draft. XL: Investigation, Supervision, Validation, Writing – review & editing. CB: Investigation, Supervision, Validation, Writing – review & editing.

## Supplementary material

Supplementary Table 1 Scientific names matching literary names of each TCM were displayed.
