# Supplementary material for: Anti-HIV activity in traditional Chinese medicine: clinical implications of monomeric herbal remedies and compound decoctions
Source: Front Med (Lausanne). 2024 Aug 8;11:1322870. doi: 10.3389/fmed.2024.1322870 (PMC11340536; doi:10.3389/fmed.2024.1322870)
Supplement: Supplementary Table 1 — Scientific names matching literary names of each TCM were displayed. [file Data_Sheet_1.docx]

| **Literary Names** | **Scientific Names** |
| --- | --- |
| Acacia | Fabaceae; Albizia lebbeck (L.) Benth. |
| Aconite | Ranunculaceae; Aconitum carmichaelii Debeaux |
| Acorus Calamus | Acoraceae; Acorus calamus L. |
| Alisma | Alismataceae; Alisma plantago-aquatica subsp. orientale (Sam.) Sam. |
| American Ginseng | Araliaceae; Panax quinquefolius L. |
| Amomum Villosum | Zingiberaceae; Wurfbainia villosa (Lour.) Škorničk. & A.D.Poulsen |
| Andrographis Paniculata | Acanthaceae; Andrographis paniculata (Burm.f.) Wall. ex Nees |
| Andrographis Pchioides | Acanthaceae; Andrographis echioides (L.) Nees |
| Angelica | Apiaceae; Angelica sinensis (Oliv.) Diels |
| Arisaema | Araceae; Arisaema erubescens (Wall.) Schott |
| Asparagus | Asparagaceae; Asparagus cochinchinensis (Lour.) Merr. |
| Astragalus | Fabaceae; Astragalus mongholicus Bunge |
| Atractylodes | Asteraceae; Atractylodes lancea (Thunb.) DC. |
| Baical Skullcap | Lamiaceae; Scutellaria baicalensis Georgi |
| Bletilla striata | Orchidaceae; Bletilla striata (Thunb.) Rchb.f. |
| Buddha's hand | Rutaceae; Citrus × limon (L.) Osbeck |
| Bupleurum | Apiaceae; Bupleurum chinense DC. |
| Caper Spurge | Capparaceae; Capparis spinosa L. |
| Cardamom | Zingiberaceae; Elettaria cardamomum (L.) Maton |
| Caterpillar Fungus | Ophiocordycipitaceae; Ophiocordyceps sinensis |
| Chinese Yam | Dioscoreaceae; Dioscorea oppositifolia L. |
| Cinnamon | Lauraceae; Cinnamomum verum J.Presl |
| Citrus Aurantium | Rutaceae; Citrus × aurantium f. aurantium |
| Codonopsis | Campanulaceae; Codonopsis pilosula (Franch.) Nannf. |
| Coix Lacryma | Poaceae; Coix lacryma-jobi L. |
| Coix seed | Poaceae; Coix lacryma-jobi var. ma-yuen (Rom.Caill.) Stapf |
| Coptis | Ranunculaceae; Coptis chinensis Franch. |
| Cortex Eucommiae | Eucommiaceae; Eucommia ulmoides Oliv. |
| Cypress | Cupressaceae; Cupressus sempervirens L. |
| Dandelion | Asteraceae; Taraxacum sect. Taraxacum F.H.Wigg. |
| Dodder | Convolvulaceae; Cuscuta chinensis Lam. |
| Dry Ginger | Zingiberaceae; Zingiber officinale Roscoe |
| Epimedium | Berberidaceae; Epimedium sagittatum (Siebold & Zucc.) Maxim. |
| Fangfeng | Apiaceae; Saposhnikovia divaricata (Turcz. ex Ledeb.) Schischk. |
| Forsythia  Fraxinus bark | Oleaceae; Forsythia suspensa (Thunb.) Vahl  Oleaceae; Fraxinus excelsior L. |
| Galla Rhois | Anacardiaceae; Rhus chinensis Mill. |
| Ganoderma | Ganodermataceae; Ganoderma lucidum |
| Gardenia | Rubiaceae; Gardenia jasminoides J.Ellis |
| Gastrodia | Orchidaceae; Gastrodia elata Blume |
| Ginseng | Araliaceae; Panax ginseng C.A.Mey. |
| Glossy Privet Fruit | Oleaceae; Ligustrum lucidum W.T.Aiton |
| Glycine | Fabaceae; Glycine max (L.) Merr. |
| Goji | Solanaceae; Lycium barbarum L. |
| Goji Berry | Solanaceae; Lycium barbarum L. |
| Goldthread | Ranunculaceae; Coptis chinensis Franch. |
| Green beans | Fabaceae; Vigna radiata (L.) R.Wilczek |
| Half Branch Lotus | Lamiaceae; Scutellaria barbata D.Don |
| Hawthorn | Rosaceae; Crataegus monogyna Jacq. |
| Hedyotis Diffusa | Rubiaceae; Scleromitrion diffusum (Willd.) R.J.Wang |
| Honeysuckle | Caprifoliaceae; Lonicera japonica Thunb. |
| Horny goat weed | Berberidaceae; Epimedium koreanum Nakai |
| Houttuynia | Saururaceae; Houttuynia cordata Thunb. |
| Isatis | Brassicaceae; Isatis tinctoria L. |
| Jujube | Rhamnaceae; Ziziphus jujuba Mill. |
| Kelp Japonica/Kelp | Phaeophyceae; Saccharina japonica |
| Knotweed | Polygonaceae; Reynoutria japonica Houtt. |
| Kudzu root | Fabaceae; Pueraria montana var. lobata (Willd.) Maesen & S.M.Almeida ex Sanjappa & Predeep |
| Licorice | Fabaceae; Glycyrrhiza uralensis Fisch. ex DC. |
| Ligusticum | Apiaceae; Conioselinum anthriscoides 'Chuanxiong' |
| Ligustrum | Oleaceae; Ligustrum lucidum W.T.Aiton |
| Lithospermum | Boraginaceae; Lithospermum officinale L. |
| Lycium Barbarum | Solanaceae; Lycium barbarum L. |
| Morinda | Rubiaceae; Morinda citrifolia L. |
| Mulberry Bark | Moraceae; Morus alba L. |
| Oldenlandia | Rubiaceae; Scleromitrion diffusum (Willd.) R.J.Wang |
| Ophiopogon | Asparagaceae; Ophiopogon japonicus (Thunb.) Ker Gawl. |
| Patchouli | Lamiaceae; Pogostemon cablin (Blanco) Benth. |
| Peach Kernel | Rosaceae; Prunus persica (L.) Batsch |
| Peony | Paeoniaceae; Paeonia lactiflora Pall. |
| Peppermint | Lamiaceae; Mentha canadensis L. |
| Peristrophe Roxburghiana | Acanthaceae; Dicliptera tinctoria (Nees) Kostel. |
| Phellodendron | Rutaceae; Phellodendron amurense Rupr. |
| Pinellia | Araceae; Pinellia ternata (Thunb.) Makino |
| Polygonatum | Asparagaceae; Polygonatum odoratum (Mill.) Druce |
| Polygonum | Polygonaceae; Polygonum aviculare L. |
| Poria  Prince Ginseng | Polyporaceae; Poria cocos(Schw.)Wolf  Caryophyllaceae; Pseudostellaria heterophylla (Miq.) Pax |
| Radix Zanthoxyli | Rutaceae; Zanthoxylum armatum DC. |
| Rehmannia | Orobanchaceae; Rehmannia glutinosa (Gaertn.) DC. |
| Rhizoma Pinelliae | Araceae; Pinellia ternata (Thunb.) Makino |
| Rhubarb | Polygonaceae; Rheum palmatum L. |
| Rhynchophylla | Rubiaceae; Uncaria rhynchophylla (Miq.) Miq. |
| Rorydalis | Papaveraceae; Corydalis edulis Maxim. |
| Rubia cordifolia | Rubiaceae; Rubia cordifolia L. |
| Safflower | Asteraceae; Carthamus tinctorius L. |
| Salvia | Lamiaceae; Salvia rosmarinus Spenn. |
| Schisandra | Schisandraceae; Schisandra chinensis (Turcz.) Baill. |
| Scrophularia | Scrophulariaceae; Scrophularia ningpoensis Hemsl. |
| Scutellaria | Lamiaceae; Scutellaria baicalensis Georgi |
| sour Jujube Kernel | Rhamnaceae; Ziziphus jujuba var. spinosa |
| Thunder god vine | Celastraceae; Tripterygium wilfordii Hook.f. |
| Trichosanthes kirilowii | Cucurbitaceae; Trichosanthes kirilowii Maxim. |
| Tripterygium Wilfordii | Celastraceae; Tripterygium wilfordii Hook.f. |
| Turmeric | Zingiberaceae; Curcuma longa L. |
| Viola | Violaceae; Viola odorata L. |
| Wheat | Poaceae; Triticum aestivum L. |
| White Atractylodes | Asteraceae; Atractylodes macrocephala Koidz. |
| White Peony | Paeoniaceae; Paeonia lactiflora Pall. |
| Wolfi Poria extensa | Polyporaceae; Poria cocos(Schw.)Wolf |
| XuanShen | Scrophulariaceae; Scrophularia ningpoensis Hemsl. |
| Yam | Dioscoreaceae; Dioscorea oppositifolia L. |
| Yuanzhi | Polygalaceae; Polygala tenuifolia Willd. |
| Zedoary | Zingiberaceae; Curcuma zedoaria (Christm.) Roscoe |
